# The power of putting a label on it: green labels weigh heavier than contradicting product information for consumers’ purchase decisions and post-purchase behavior

**DOI:** 10.3389/fpsyg.2015.01392

**Published:** 2015-09-23

**Authors:** Ulf J. J. Hahnel, Oliver Arnold, Michael Waschto, Liridon Korcaj, Karen Hillmann, Damaris Roser, Hans Spada

**Affiliations:** ^1^Department of General Psychology, Albert Ludwigs University FreiburgFreiburg, Germany; ^2^Consumer Decision and Sustainable Behavior Lab, Department of Psychology, University of GenevaGeneva, Switzerland; ^3^Department of Personality and Social Psychology, Otto von Guericke UniversityMagdeburg, Germany

**Keywords:** product labeling, ecological motives, perceived matching, self-congruity, behavioral consistency, moral self-regulation, conservation (ecological behavior)

## Abstract

Green products are appealing. Thus, labeling products as environmentally friendly is an effective strategy to increase sales. However, the labels often promise more than the products can actually deliver. In the present research, we examined the expectation that consumers with high ecological motivation have strong preferences for green-labeled products – even when presented product information contradicts the label’s image. This unsettling hypothesis is grounded in the labels’ potential to create a cognitive match between the labeled product and consumers’ motives. For labels indicating environmental friendliness (green product labels), this link should be strongest when consumers’ ecological motivation is high. Findings in a series of three experiments support our assumption, showing that consumers with high ecological motivation had strong preferences (i.e., product evaluations, purchase intentions, and simulated purchase decisions) for green-labeled products as compared to consumers with low ecological motivation (Studies 1–3). Crucially, these preferences were robust, despite contradicting environmental product information (Studies 1 and 2). We extended our findings by additionally examining the impact of product labels and motivation on moral self-regulation processes. This was established by assessing participants’ pro-social behavior after the purchase task: participants with high ecological motivation acted, consistent with their motives, more pro-socially in post-decision occasions. In accordance with moral cleansing effects, pro-social behavior was intensified after purchasing conventional products (Studies 2 and 3). Green labels protected participants with high ecological motivation from moral threats due to the purchase, thus making pro-social behavior less likely. Findings suggest that highly ecologically motivated consumers are most susceptible to green labels, which may override detailed product information.

## Introduction

Green sells. Citizens all over the world have become more aware of local and global environmental issues and environmentally friendly behavior has fundamentally increased in the last decades (e.g., Reynolds et al., 2010; Otto and Kaiser, 2014). Hence, marketing products as “green” has become an important retail strategy: an increasing amount of products on the market are labeled as environmentally friendly (Solomon, 2013).

The increasing relevance of pro-environmentalism and corporate social responsibility (Podnar and Golob, 2007) provides strong incentives for producers to market conventional (unsustainable) products as environmentally friendly. Product labels may facilitate shallow processing of information that primarily relies on superficial cues rather than on detailed information. Specifically, green labels^1^ may lead consumers to automatically infer that the products are environmentally friendly (Gruber et al., 2014) even when they are not. In this vein, previous research has shown that labeling products as “eco-friendly” (or organic) strongly impacts purchase-relevant judgments such as perceptions of the product’s environmental attributes and beyond (e.g., Lee et al., 2013; Sörqvist et al., 2013, 2015b).

Classic information processing models predict that shallow information processing based on external cues such as labels is especially likely among consumers with low motivation (Petty and Cacioppo, 1986). That is, in the realm of sustainable purchases, consumers who describe themselves as less ecologically motivated should rely on green product labels, whereas highly motivated consumers are expected to comprehensively attend to relevant product information. According to this approach, lowly ecologically motivated consumers should be more prone to erroneous product inferences due to inadequate or ambiguous product labeling as compared to highly motivated consumers. Interestingly, recent research on product labeling suggests that in particular consumers with high ecological motivation are susceptible to product labels. For instance, highly ecologically motivated consumers were more likely to evaluate coffee or bananas as more tasty as compared to lowly motivated consumers when the product was labeled as eco-friendly (i.e., green; Sörqvist et al., 2013, 2015b). These label effects also impacted objectively measured task performance. Again, effects were more pronounced among participants with high ecological motivation (Sörqvist et al., 2015a).

The present research extends previous research on green label effects by investigating the robustness of the labels’ influence on purchase decisions (see also Sörqvist et al., 2015b). To this end, we provided product information in addition to green labels either supporting or contradicting the label. We assume that even attending to product information does not necessarily diminish the impact of green labeling on purchase decisions – especially when consumers’ ecological motivation is high. Our assumption is grounded in the label’s potential to create a match between the labeled product and highly ecologically motivated consumers prior to acquisition of detailed information.

We argue that green labels are effective by signaling to consumers with high ecological motivation that the labeled product meets their motivation and goals. Previous research has shown that ecological motivation is grounded in moral aspirations (Feinberg and Willer, 2013). Taking the connection between the environmental and moral domain into account, we assume that green labels’ impact can conceivably extend to the moral domain. That is, the influence of the established product-consumer link via the green label is expected to be twofold: it should make a purchase more likely and keep the moral self in balance – even in the face of contradicting environmental product information. In the present research, we examined this match between the labeled product and highly ecologically motivated consumers at the moral level by assessing consumers’ post-purchase behavior. Specifically, we investigated the extent to which participants compensate for potential threats to their moral self-concept due to the purchase (product labels: green or conventional) by acting pro-socially in post-purchase occasions.

In the following sections, we first review previous research on the impact of product labeling, ecological motivation, and the interaction of both on purchase-relevant information processing, serving as a basis of our conceptual framework. Second, we report research on the effects of green product labeling on moral self-regulation processes. Finally, we introduce ecological motivation as an important moderator in moral self-regulation processes.

## Impact of Labels on Motivated Information Processing

Previous research on product labeling illustrates that labeling products as pro-environmental or ethical are effective means to influence consumers’ choices (Thøgersen et al., 2012; Lee et al., 2013; Sörqvist et al., 2013, 2015b; Bradu et al., 2014; Wiedmann et al., 2014). Such green labeling communicating environmental friendliness can be realized by various means, be it by colors, symbols, or official seals.

We argue that green labels increase purchase intentions by making consumers with high ecological motivation perceive the product as compatible with their ecological motives. Supporting this assumption, previous research found that ecological motivation is positively related to purchase frequency and the willingness to pay for the product when it was labeled as green (Grankvist and Biel, 2007; Lee et al., 2013; Sörqvist et al., 2013, 2015b; Wiedmann et al., 2014). The link between the labeled product and consumers with high ecological motivation is usually established prior to the acquisition of more detailed product information. Negative product information, which contradicts this formed impression, may evoke a perceived cognitive inconsistency in highly ecologically motivated consumers. It is an established finding that individuals strive for consistency at the behavioral and cognitive level (e.g., Festinger, 1957). Belief harmonization theory (Dunning, 2007) emphasizes that consumers align their beliefs about a certain product to retain a positive self-concept of a rational and coherent person. That is, when consumers initially approve of a given product, they will tend to cognitively harmonize beliefs about it, for example by altering the personal relevance of certain product attributes. Further strategies to retain consistent positive product beliefs encompass the development of counterarguments in response to negative information as well as the denegation of the diagnosticity of negative information (Ahluwalia et al., 2000; Jain and Maheswaran, 2000; Ahluwalia, 2002). With respect to environmentally relevant consumption, all of these processes render consumers with high ecological motivation more likely to amplify positive and attenuate negative information about green-labeled products. These altered perceptions are expected to result in stable positive purchase intentions regarding green-labeled products for consumers with high ecological motivation.

Previous research supports the assumption that consumers with high ecological motivation tend to make stable positive judgments toward environmentally friendly products. Ecological motives were found to strongly and positively impact purchase intentions toward sustainable products, such as electric vehicles (EVs; Hahnel et al., 2014a). Effects were mediated by consumers’ cognitions about the product: ecological motives were positively related to the perceived match between presented product attributes and participants’ individual demand (perceived matching). This perception, in turn, was related to participants’ purchase intentions: participants perceiving that EV attributes more closely matched their individual demand reported stronger purchase intentions. These findings are in line with research showing that highly ecologically motivated consumers tend to judge products as superior and are more willing to pay for them when the products are labeled as environmentally friendly (e.g., Lee et al., 2013; Sörqvist et al., 2013, 2015b). Remarkably, the positive relationship between ecological motivation and willingness to pay seems to be stable, thus hardly affected by other personal preferences such as taste (Sörqvist et al., 2013). In the present research, we aimed to extend findings on the robustness of the positive relationship between ecological motivation and green-labeled products by providing product information in addition to the label. Crucially, we were interested in whether highly ecologically motivated consumers still report positive product evaluations and high purchase intentions even in the face of contradicting product information.

Findings regarding the impact of motivation on product perceptions are in accordance with self-image congruence models, emphasizing that consumers use products to express their personality (Sirgy, 1982). Following these models, self-congruity refers to the perceived match between a typical product user and one’s own personality traits. Hence, self-congruity covers a variety of personality traits that are represented by a given product. A new pair of running shoes, for example, may represent that one is sportive, stylish, and aﬄuent. Self-congruity impacts cognitions about the product’s functional characteristics (Sirgy et al., 1991). Namely, the more consumers believe that a given product matches their personality, the more they perceive the product’s attributes as superior (biasing effect).

In the present research, we incorporated participants’ perceived matching of product attributes with their individual demand to assess whether these perceptions mediate effects of ecological motivation on purchase intentions toward green-labeled products (Study 1). Moreover, we extended findings on perceived matching by measuring participants’ self-congruity prior to acquisition of product information (Study 2).

## Impact of Labels on Moral Self-Regulation

Green labels seem to also affect behavior subsequent to the purchase situation. Choosing green-labeled products, for example, may stimulate anti-social behavior. In previous research, participants who chose green products tended to show more anti-social behavior in subsequent moral tasks (Mazar and Zhong, 2010). These findings can be interpreted in terms of moral self-regulation. Individuals aim at maintaining a positive moral self-concept, thus constantly engaging in an unconscious moral self-regulation process (Khan and Dhar, 2006; Sachdeva et al., 2009). This moral self-regulation implies that individuals are motivated to re-establish their moral self-concept after actions that are perceived as immoral (moral cleansing) and feel licensed to act less moral after their moral image has been boosted (moral licensing).

Recent research has tried to identify moderator variables, which determine whether or not moral self-regulation will occur (for an overview see: Blanken et al., 2015). While some have argued that an individual’s current mind-set (i.e., rule-based vs. outcome-based) moderates the influence of previous moral behavior on subsequent actions (Cornelissen et al., 2013), others have found that the occurrence of a moral licensing effect may depend on individuals’ chronic goal commitment (Effron et al., 2009; Merritt et al., 2010). Specifically, in the domain of racial discrimination, moral self-regulation was exclusively found for participants high in racial prejudice (i.e., low commitment to egalitarian goals, Effron et al., 2009). Contrarily, self completion theory (e.g., Wicklund and Gollwitzer, 1981) posits that attaining self-relevant goals (high goal commitment) results in a state of identity-goal completeness, which tempers goal striving. Supporting this assumption, positive feedback on purchasing environmentally friendly products led to less recycling as compared to neutral or negative feedback of participants with high goal commitment (Longoni et al., 2014). Likewise, we argue that consumers’ ecological motivation takes on a moderating role in the realm of sustainable purchases. A product label should be particularly relevant when being environmentally friendly has a strong relevance for the self. Hence, moral self-regulation is expected to particularly occur for highly ecologically motivated consumers.

These expectations are grounded in the close relationship between motives and the self. Being motivated to or actually acting pro-environmentally may substantially shape a person’s self-concept (e.g., Verplanken and Holland, 2002; Van der Werff et al., 2013, 2014). In previous research, activating ecological motives only resulted in environmentally friendly consumption when the motives were central to the person’s self (Verplanken and Holland, 2002; Hahnel et al., 2014b). Moreover, recent research emphasized the role of (anticipated) emotions as drivers of pro-environmental behavior (Mallett, 2012; Onwezen et al., 2013, 2014a,b; Brosch et al., 2014). The elicitation of emotions is, in turn, determined by the personal relevance of the respective behavior for the person (Brosch et al., 2014). Correspondingly, effects of anticipated emotions such as guilt and pride on environmentally friendly behavior seem to be primarily evident for consumers with strong ecological motivation (Mallett, 2012; Onwezen et al., 2014a,b).

The influence of emotions on environmentally friendly behavior (e.g., Mallett, 2012) suggests that, for highly ecologically motivated consumers, environmentally friendly behaviors constitute an important aspect of the moral self-concept. This assumption is supported by research showing that environmentally friendly behavior is largely guided by moral aspirations (Feinberg and Willer, 2013). We argue that for highly ecologically motivated consumers, green labels signalize that the labeled product matches their moral self-concept. That is, the established product-consumer link via the green label is expected to have two important implications: first, it should make a purchase more likely and, second, attenuate potential moral threats due to it – even in the face of contradicting product information.

Summarizing, high ecological motivation implies a high relevance of environmentally friendly behavior for the person’s self. Hence, inadequate behavior within the environmental domain (e.g., purchasing conventionally labeled products) should be more likely to endanger the moral integrity of a highly ecologically motivated person, thus stimulating moral self-regulation processes. By contrast, the link between green-labeled products and highly ecologically motivated consumers should protect them from a moral threat.

## Current Research

In a series of three experiments, we aimed to shed light on the impact of a variety of green labels on purchase and post-purchase behavior. The selected products had either an implicit green label (i.e., EVs, Study 1) or embodied explicit (physical) labels (Studies 2 and 3). In Study 1, the green label was based on the product’s reputation as being environmentally friendly. We applied EVs as target products, which have a strong pro-environmental image (Griskevicius et al., 2010; Noppers et al., 2014) rather than a physical green label. In Study 2, we applied a physical green label by including the word “nature” in the target product’s name and a picture (of two leafs) on the product (for more details on the applied label see Section “Product Label”). Note that this label was not based on official ecological certification. In Study 3, in accordance to Mazar and Zhong (2010), we applied a variety of officially certified physical green labels. This approach allowed for investigating the impact of a variety of green labels (certified and non-certified), thus providing knowledge about the relevance of certain label characteristics for purchase decisions.

We hypothesized that consumers with high ecological motivation in particular are likely to make positive product inferences when products are labeled as green. We assumed the positive inferences would be based on the match between the green label and said consumers’ motives. This match should in turn have fundamental implications for consumers’ purchase decisions and post-purchase behavior. Green labels were expected to increase purchase intentions, override negative product information, and protect consumers from threats to their moral self-concept when consumers’ ecological motivation is high.

In Study 1, EVs were selected as the target product – a technology with a strong sustainable image (e.g., Griskevicius et al., 2010; Noppers et al., 2014). We measured participants’ ecological motivation and experimentally varied environmental product information (positive/neutral/negative). We assessed participants’ purchase intentions and their perceived matching of the product’s attributes with their individual demand (perceived matching: Hahnel et al., 2014a,b) as outcome variables. The experimental variation of product information allowed for examining whether the anticipated link between EVs and participants with high ecological motivation resulted in positive product preferences, even in the presence of contradicting product information.

In Study 2, we examined whether the impact of ecological motivation and product information was indeed subject to the product’s label. We experimentally varied the product’s physical label (green/conventional) and measured participants’ ecological motivation. The influence of the product’s label was assumed to substantially differ depending on the degree of participants’ ecological motivation. Furthermore, we aimed to extend findings from Study 1 on perceived matching by incorporating the concept of self-congruity (Sirgy et al., 1991). While perceived matching assesses a perceived match between the product’s attributes and one’s individual demand (after acquisition of information), including self-congruity allowed to examine the effects of an initial product-self matching on the personality level (prior to acquisition of information).

In Studies 2 and 3, we went beyond the actual purchase situation by additionally assessing participants’ pro-social behavior subsequent to the purchase task. We hypothesized that the established link between the product and highly motivated consumers also protects them from moral threats due to the purchase. Thus, a green label should make a given purchase more likely when ecological motivation is high, but also attenuate potential moral threats due to the purchase. Conventional labels, in contrast, should not provide an opportunity to preserve the moral self-concept. Under this condition, highly motivated participants should intensify their pro-social behavior in subsequent occasions to compensate for moral threats. As environmental behavior should be morally less relevant for participants with low ecological motivation, these moral self-regulation processes were expected to be in particular evident for highly motivated consumers. To gain deeper insights into the scope of moral self-regulation, we examined pro-social behavior within the same (environment, Study 3) as well as in a different pro-social domain (child charity, Study 2).

## Study 1

In Study 1, we investigated effects of green labels on purchase intentions toward a product with a strong sustainable image (EVs; e.g., Noppers et al., 2014). Thus, we applied a target product with an implicit green label rather than a physical label (cf. Studies 2 and 3). We were interested in the interaction of ecological motivation and product information as we hypothesized highly ecologically motivated consumers to consistently favor products that signal to match their ecological motives. To this end, we experimentally varied information about product attributes related (EV environmental attributes: positive/neutral/negative) and unrelated to ecological motivation (EV purchasing price: moderate/high). In line with previous research, participants’ ratings of how well product attributes match their individual demand were used to assess product attribute evaluations (perceived matching; see e.g., Hahnel et al., 2014a).

Previous research found that participants with high ecological motivation were affected by negative as well as positive environmental product information in terms of purchase decisions, while participants with moderate ecological motivation were in particular influenced by negative information (Grankvist et al., 2004). In Study 1, we extended this research by investigating effects of environmental product information regarding a product with an implicit green label (EVs).

We experimentally varied EV purchasing price, in addition to the environmental attribute information. Previous research has shown that high ecological motivation is accompanied by a higher willingness to pay for labeled products (e.g., Sörqvist et al., 2013). In addition, activation of ecological motives resulted in lower price sensitivity toward EVs (Hahnel et al., 2014b). That is, participants were less affected by EV purchasing price changes when ecological motives were activated. Taking these previous findings into account, in Study 1, we examined whether highly ecologically motivated participants were less sensitive to the experimental variations in EV purchasing price as compared to lowly motivated participants.

We hypothesized that participants’ ecological motivation would positively affect their perceived matching with presented EV attributes (Hypothesis 1). Additionally, we expected that participants’ product attribute evaluations would be shaped by provided product information (Hypothesis 2). Moreover, we assumed ecological motivation and environmental information to interact: participants with high ecological motivation should be more likely to have high purchase intentions toward EVs, even in the presence of negative environmental product information (direct pathway, Hypothesis 3). Finally, we hypothesized that perceived matching would mediate the impact of ecological motivation on purchase decisions (indirect pathway, Hypothesis 4).

### Method

#### Participants and Design

Data were collected from a representative sample of 269 participants (129 female), recruited by a German market research institute. Participants received a compensation of 1.5€ (USD 1.97) for completing the online study. Of the 269 participants, 22 did not report the desired target price of their next car (cf. covariates) and thus were excluded from the main analyses.

Participants voluntarily agreed to take part in the studies presented here and had the opportunity to withdraw from participation at any stage of the experiments. The studies were conducted in accordance with the ethical standards described by the German Science Foundation (DFG, 2015). These guidelines exempt research on healthy humans from ethical review when the research neither involves personal risks nor high physical or emotional stress. As the present research entirely meets these ethical requirements, no formal approval was requested.

The study was based on a 3 (EV environmental information: positive/neutral/negative) × 2 (EV purchasing price information: moderate/high) experimental between-subjects design.

#### EV Attribute Information

Electric vehicle attribute information was adapted from previous studies on purchase decisions in the realm of EVs (Hahnel et al., 2014a,b). Provided information on EVs was based on extensive literature and internet research and reflected an EV attribute standard that was slightly above the state of the art at the time of data collection (May, 2013). EV attribute information was divided into subsections of 31–98 words each addressing a product attribute: environmental friendliness, purchasing price, performance, range, charging time, and charging costs. In addition to the specific subsections, the same summary of all attributes was presented on each information subsection page (cf. Appendix B for presented EV attributes).

##### EV environmental information variation

Electric vehicle environmental information focused on the fact that, although EVs’ direct CO_2_-emissions are zero, the total CO_2_-emissions caused by EVs strongly depend on how the electricity is generated as well as on the efficiency of the electric engine (Heider et al., 2009).

At the beginning of this section, it was stated that EVs have either enormous advantages (positive condition), some disadvantages (negative condition), or both advantages as well as disadvantages (neutral condition) in terms of environmental attributes as compared to conventional vehicles. Statements were justified by EVs’ total CO_2_-emissions. We provided information about the average CO_2_-emissions of a conventional vehicle (175 g CO_2_/km), informing that EVs’ CO_2_-emissions were either lower (positive condition: 0 g CO_2_/km, renewable energy sources), similar (neutral condition: 175 g CO_2_/km, neutral statement), or higher (negative condition: 225 g CO_2_/km, primarily fossil energy sources) than those of a conventional vehicle. Differences between EVs’ and conventional vehicles’ CO_2_-emissions were additionally emphasized with a bar chart depicting the amount of CO_2_-emissions (in g CO_2_/km) of both vehicles next to each other.

Finally, we varied information about the environmental impact of EVs due to the production and disposal of the vehicles’ batteries and materials. This was described as non-intrusive (positive condition), unpredictable (neutral condition), or intrusive (negative condition).

##### EV purchasing price variation

Information regarding the purchasing price of EVs was presented in a separate subsection. Price information was based on a middle class car [moderate price: 25,000€ (USD 32,860); high price: 35,000€ (USD 46,000)]. Additionally, we provided information about the extra costs compared to a conventional vehicle [moderate price: 5,000€ (USD 6,570); high price: 15,000€ (USD 19,720)].

##### Control questions

We included a set of four control questions about the presented product information (e.g., CO_2_-emission values of EVs and conventional vehicles) to ensure that participants actually read and understood the provided information. When participants answered one of the control questions incorrectly, they were returned to both information subsections and had the opportunity to repeat the test. Participants were automatically screened out when they did not pass this second attempt.

##### Manipulation check

We examined whether our experimental variations of EV environmental information and purchasing price were successful in that they influenced perceptions of the respective product attributes. Hence, variations in EV environmental attribute information were expected to influence participants’ perceptions of EVs’ environmental attributes, while purchasing price variation should influence participants’ perceptions of EV purchasing price. Effects of the two information factors should be limited to the respective product attributes, as information regarding other EV attributes was kept constant across information conditions. To examine whether effects of information variation were indeed limited to the intended product attributes, we also assessed attribute perceptions of EV charging time, charging costs, range, and performance.

The six items consisted of a statement “The [respective EV attribute] of an EV is…” that was answered on a 6-point Likert scale ranging from 1 (*very low/very bad*) to 6 (*very high/very good*). Thus, for example, participants indicated the extent to which they perceive the presented EV purchasing price (25,000€ or 35,000€) as rather low or high (EV purchasing price perception). A multivariate analysis of variance (MANOVA) including EV environmental information and EV purchasing price as factors and the six items measuring product attribute perceptions as dependent variables revealed multivariate main effects of environmental information [*F*(12,518) = 10.71, *p* < 0.001, $\eta_{p}^{2}$ = 0.20] and EV purchasing price [*F*(6,258) = 5.71, *p* < 0.001, $\eta_{p}^{2}$ = 0.12] but no significant interaction of the two factors [*F*(12,518) = 0.62, *p* = 0.826, $\eta_{p}^{2}$ ≤ 0.01] on perceptions of product attributes. Specifically, environmental information significantly influenced perceptions of EVs’ environmental attributes [*F*(2,263) = 69.53, *p* < 0.001, $\eta_{p}^{2}$ = 0.35] but not on the remaining attributes [0.49 ≤*F*(2,263) ≤ 1.3, *p* > 0.05, $\eta_{p}^{2}$ < 0.01]. *Post hoc* tests indicated that environmental information conditions strongly differed in terms of perceived EV environmental attributes (Sidak, *p* < 0.001, for all differences). Additionally, results showed a main effect of EV purchasing price on participants’ purchasing price perceptions [*F*(1,263) = 33.03, *p* < 0.001, $\eta_{p}^{2}$ = 0.11] but no difference in terms of the remaining EV attributes [0.69 ≤*F*(1,263) ≤ 3.87, *p* > 0.05, $\eta_{p}^{2}$ < 0.02]. Findings confirmed that the experimental variations were strongly effective in influencing perceptions regarding the respective attribute while not affecting those regarding other product attributes (i.e., EV charging time, charging costs, range, and performance).

Moreover, to check for a difference between information conditions in terms of perceived quality of provided information, we conducted a second MANOVA with information conditions as factors and participants’ ratings of how informative, objective, and credible the information was as dependent variables. Multivariate tests showed that there was neither a difference in participants’ ratings between EV environmental information conditions [*F*(6,524) = 0.54, *p* = 0.78, $\eta_{p}^{2}$ < 0.01], nor between EV purchasing price conditions [*F*(3,261) = 0.52, *p* = 0.67, $\eta_{p}^{2}$ < 0.01].

#### Measurements

Ecological motivation, perceived matching, and purchase intention were formatted as 6-point scales (1 – *strongly disagree* to 6 – *strongly agree*; cf. Appendix A for items). Scores were computed by calculating the mean value of the respective items^2^.

##### Ecological motivation

The strength of ecological motives was measured with four items based on a validated motive scale, assessing motives in the domain of car-use (Hahnel et al., 2014a). Participants rated the extent to which they agreed with the presented statements on environmentally friendly mobility behavior (Cronbach’s α = 0.93). To veil our interest in ecological motivation, we included 14 additional motive items not related to the environment (e.g., freedom, hedonism, costs).

##### Perceived matching with product attributes

Product attribute evaluation was measured using perceived matching (Hahnel et al., 2014a). Adopted items assessed the extent to which participants perceived that specific EV attributes (EV purchasing price, environmental attributes, CO_2_-emissions, charging time, charging costs, range, and performance) met their individual demand in the realm of car-use (Cronbach’s α = 0.73).

##### Purchase intention

Intention to purchase an EV was measured with three items (Cronbach’s α = 0.93).

##### Covariates

We controlled for the price participants sought to pay for their next car, since this variable strongly impacted perceptions of EV attributes in previous research (Hahnel et al., 2014b). Thus, the general willingness to pay for a car was expected to determine consumers’ individual demands on the product and thus their product evaluations – in particular their perceived matching ratings. This covariate was *z*-standardized prior to analysis.

As we provided information about EVs and their attributes, we controlled for participants’ prior knowledge about EVs by means of one item that was formatted on a 6-point scale ranging from 1 (*I know nothing about EVs*) to 6 (*I am an EV expert*).

#### Procedure

Participants first reported demographics, prior knowledge about EVs, and their ecological motivation. Next, participants received information about EV attributes and answered the control questions. Then, participants answered perceived matching, purchase intention, and manipulation check items. Finally, participants were debriefed and thanked.

### Results and Discussion

We conducted a conditional process analysis including ecological motivation as the independent variable, perceived matching as the mediator, and purchase intention as the dependent variable. We specified the paths from ecological motivation to perceived matching and from ecological motivation to purchase intention to be moderated by the experimentally varied factors. As the experimental factor EV environmental information was three-categorical, we computed two dummy variables (EV negative and neutral environmental information: present/absent). The EV positive information condition served as the reference group (for methodology see: Hayes, 2013, 2015).

**FIGURE 1:**
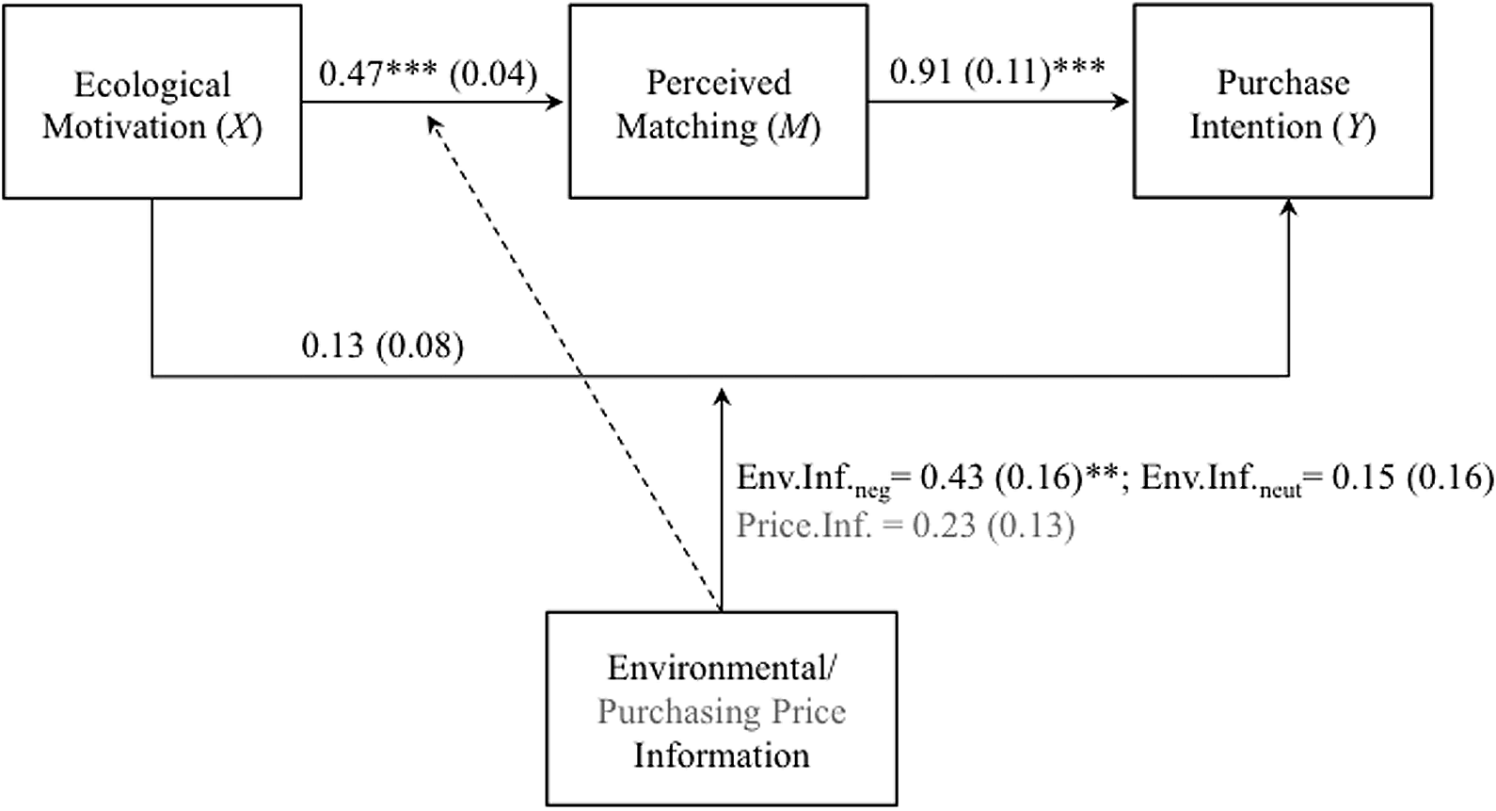
**Conditional process model testing the relationship between ecological motivation (*X*) and purchase intention (*Y*) via perceived matching (*M*) in Study 1.** Paths from ecological motivation to perceived matching and purchase intention were specified to be moderated by environmental and purchasing price information. Unstandardized coefficients are depicted on the arrows, standard errors in parentheses. Main effects of the moderators and covariates are not displayed to retain clarity. ^∗∗∗^*p* < 0.001, ^∗∗^*p* < 0.01.

As seen in **Figure 1**, ecological motivation affected perceived matching and purchase intentions. Direct effects of ecological motivation on purchase intentions were moderated by EV environmental attribute information. Specifically and in line with Hypothesis 1, there was a highly significant positive effect of ecological motivation on the perceived matching score (*a_1_* = 0.47, 95% CI [0.39, 0.55], *t* = 12.03, *p* < 0.001). Additionally, confirming Hypothesis 2, experimental variations in terms of EV environmental and purchasing price information influenced perceived matching scores (negative environmental information: *a_2_* = –0.23, 95% CI [–0.44, –0.02], *t* = –2.16, *p* = 0.03; neutral environmental information: *a_3_* = –0.10, 95% CI [–0.31, 0.11], *t* = –0.94, *p* = 0.35; purchasing price: *a_4_* = –0.25, 95% CI [–0.42, –0.08], *t* = –2.85, *p* < 0.01). That is, negative EV environmental attributes resulted in lower perceived matching ratings as compared to positive environmental information (Δ*M* = 0.23), while there were no differences between the remaining EV environmental information conditions (Δ*M_neg-neut_* = 0.13; Δ*M_neut-pos_* = 0.10). Higher EV purchasing price information resulted in lower perceived matching ratings than moderate price information (Δ*M* = 0.25). Neither the interaction of ecological motivation with EV purchasing price information, nor with the EV environmental information variables on perceived matching were significant (*a_5-7_* ≤ –0.12, 95% CI [≥–0.31, ≤0.10], *t* ≥ –1.25, *p* > 0.05). Reported next car’s target price was negatively related to perceived matching scores (*a*_8_ = –0.12, 95% CI [–0.20, –0.03], *t* = –2.68, *p* < 0.01) while participants’ prior EV knowledge was positively related to perceived matching (*a_9_* = 0.14, 95% CI [0.06, 0.22], *t* = 3.37, *p* < 0.001). The reported next car’s target price did not significantly influence purchase intention (*c*_8_’ = 0.06, 95% CI [–0.08, 0.21], *t* = 0.84, *p* = 0.40) but prior knowledge did (*c*_9_’ = 0.21, 95% CI [0.07, 0.34], *t* = 3.09, *p* < 0.01). In total, 43% of the mediator’s variance was explained by the model [*F*(9,237) = 19.56, *p* < 0.001].

As expected, there was a strong effect of perceived matching ratings on purchase intention (*b_1_* = 0.91, 95% CI [0.70, 1.13], *t* = 8.47, *p* < 0.001), showing that perceiving EV attributes as matching one’s individual motives was related to higher purchase intentions. There were no direct effects of ecological motivation and the experimental information conditions on purchase intention (*c_1-4_’* ≤ 0.13, 95% CI [≥–0.58, ≤0.44], *t* ≤ 1.52, *p* > 0.05). Also, the interaction of ecological motivation and purchasing price information was not significant (*c*_5_’ = 0.23, 95% CI [–0.13, 0.48], *t* = 1.73, *p* = 0.08). In line with Hypothesis 3, there was a significant interaction of ecological motivation with negative EV environmental information (*c_6_’* = 0.43, 95% CI [0.11, 0.75], *t* = 2.65, *p* < 0.01) but not with neutral EV environmental information (*c_7_’* = 0.15, 95% CI [–0.15, 0.46], *t* = 0.99, *p* = 0.32). Including both interaction terms significantly increased the explained variance of the dependent variable model [*F*(2,236) = 3.58, *p* = 0.03, Δ*R^2^* = 0.02], showing that the impact of ecological motivation on purchase intentions was moderated by the provided environmental information.

**FIGURE 2:**
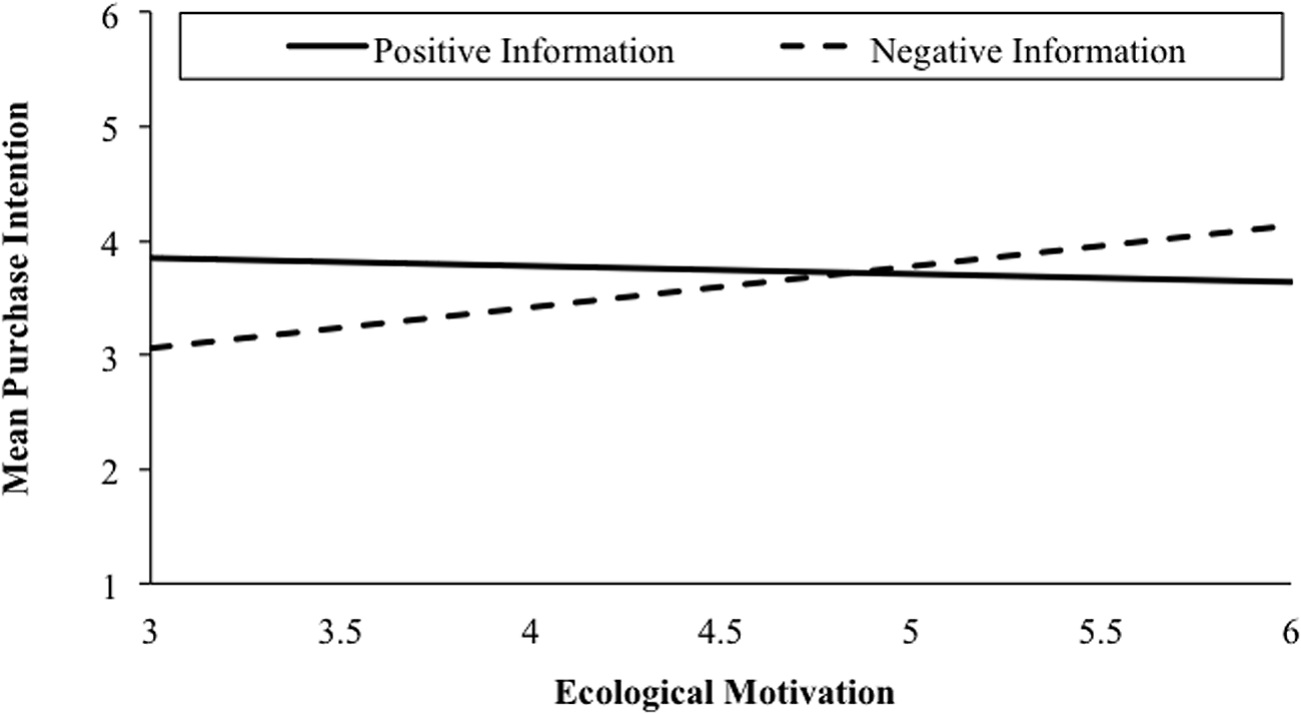
**Conditional direct effects of ecological motivation on purchase intentions as a function of experimentally varied environmental information (positive and negative conditions only) in Study 1**.

Subsequently, we calculated conditional direct and indirect effects of ecological motivation on purchase intentions^3^. Bias-corrected bootstrap confidence intervals for the conditional indirect effect of ecological motivation on purchase intention via perceived matching (*ab*_|E.Inf = *positive*_ = 0.50; *ab*_|E.Inf = *neutral*_ = 0.41; *ab*_|E.Inf = *negative*_ = 0.39) based on 5.000 bootstrap samples excluded zero in all EV environmental information conditions (positive: 0.37–0.67; neutral: 0.24–0.62; negative: 0.26–0.55). Hence, independent of provided information regarding EVs’ environmental attributes, ecological motivation was positively associated with perceiving EV attributes as more matching. Perceived matching, in turn, was related to higher purchase intentions toward EVs (cf. Hypothesis 4). As depicted in **Figure 2**, conditional direct effects revealed a positive relationship between ecological motivation and purchase intentions when EV environmental information was negative (*c*_1_’|_E.Inf = *negative*_ = 0.36, 95% CI [0.11, 0.61], *t* = 2.83, *p* < 0.01) but no significant direct effects when EV environmental information was positive (*c*_1_’|_E.Inf = *positive*_ = –0.07, 95% CI [–0.32, 0.17], *t* = –0.58, *p* = 0.56) or neutral (*c*_1_’|_E.Inf = *neutral*_ = 0.08, 95% CI [–0.16, 0.32], *t* = 0.68, *p* = 0.50). The model explained 43% of variance in the dependent variable [*F*(10,236) = 18.20, *p* < 0.001].

In summary, participants with high ecological motivation showed strong purchase intentions toward EVs. The indirect pathway via perceived matching shows that participants with high ecological motivation perceived EV attributes as more matching with their individual demand. These positive product beliefs partially mediated the impact of ecological motivation on purchase intention.

Electric vehicle purchasing price affected perceived matching scores in that a higher purchasing price was related to lower perceived matching scores. There was neither an interaction of ecological motivation and purchasing price on perceived matching scores, nor on purchase intentions. This result showed that, in the present research, there were no differences in terms of sensitivity toward EV purchasing prices between participants with high and low ecological motivation. We suggest future research to apply larger price ranges than in Study 1 (25,000€ and 35,000€), to gain more knowledge about the impact of ecological motivation on price sensitivity toward green-labeled products (see e.g., Hahnel et al., 2014b).

In contrast to purchasing price information, EV environmental information interacted with ecological motivation. The direct pathway from ecological motivation to purchase intention illustrates that ecological motivation had an additional positive direct effect on purchase intentions when EV environmental information was negative. That is, highly ecologically motivated participants even favored EVs when the provided environmental product information was negative. In previous research, highly ecologically motivated participants tended to take both, negative as well as positive environmental product information into account when making purchase decisions (Grankvist et al., 2004). We assume that EVs’ green image stimulated a cognitive match between the product and highly ecologically motivated consumers prior to information acquisition. This match resulted in stable purchase preferences for EVs, even when contradicting information was subsequently depicted. Under this condition, less environmentally motivated consumers dismissed the product.

## Study 2

In Study 2, we experimentally varied the product’s physical label to examine if the unshakable positive relationship we found between ecological motivation and purchase decisions is indeed limited to products with a green label. We used a suntan lotion as the target product, which allowed to vary the product’s physical label (green/conventional) while keeping additional product attributes constant. We applied a physical green label by including the word “nature” in the target product’s name and a product picture (of two leafs) on the product. Thus, this label was not based on official ecological certification. In contrast to Study 1, we provided all participants with negative environmental information.

We also aimed to extend findings on perceived matching by measuring participants’ perceived self-congruity (e.g., Sirgy, 1982). In Study 1, effects of ecological motivation on purchase intentions were partially mediated by perceived matching (indirect pathway), which was assessed after information had been provided. In Study 2, we measured self-congruity before information acquisition by assessing the perceived similarity between a typical product user and one’s own personality traits. Hence, self-congruity refers to a general product-consumer matching on the personality level, incorporating multiple consumer traits. In previous research, self-congruity was related to more positive evaluations of product attributes and higher purchase intentions (biasing effect, e.g., Sirgy et al., 1991). In contrast to self-congruity, which covers multiple personality traits, we expected the match created by the green label to be specific to consumers’ ecological motivation. Green labels address consumers’ ecological motives by signaling to them that the product meets their ecological motivation and goals. In Study 2, we controlled for self-congruity to gain more knowledge about effects of ecological motivation in green-labeled product purchase decisions. We expected ecological motivation and product label to explain incremental variance in addition to self-congruity, as green label effects are grounded in consumers’ motivation to act environmentally friendly and in the labels’ ability to address this specific motivation.

We aimed to further extend the findings on purchase intentions from Study 1 by additionally examining participants’ pro-social behavior subsequent to the purchase task. Specifically, we were interested in whether the match established by the green label also makes a given purchase more morally justifiable for highly motivated participants. We assessed participants’ pro-social behavior using a donation paradigm after they envisioned having purchased the target product (e.g., Sachdeva et al., 2009). We assumed the donation would serve as a means to compensate for potential moral threats due to the purchase by showing pro-social behavior in subsequent occasions (moral cleansing). This allowed investigating whether green labels indeed have a protective function, preserving consumers with high ecological motivation from threats to their moral self-concept, even in the presence of contradicting product information. As post-purchase behavior referred to a different pro-social domain than the environment (child charity), we examined the extent to which moral cleansing effects occur when the immoral and compensatory behaviors belong to different domains (see also: Sachdeva et al., 2009; Blanken et al., 2015).

Based on findings from Study 1, we hypothesized an interaction of ecological motivation and product label on product evaluation after (negative environmental) information presentation: participants with high ecological motivation should comparatively decrease their product evaluations due to negative environmental information when the product is labeled as conventional, but not when the product has a green label (Hypothesis 5). In terms of the subsequent moral choice task, we expected that participants donate more money in the conventional as compared to the green label condition (Hypothesis 6; cf. Mazar and Zhong, 2010). Crucially, we again expected ecological motivation and product label to interact: participants with high ecological motivation allocated to the conventional label condition should donate the highest amount (Hypothesis 7). Under this condition, the threat to participants’ moral self-concepts and thus their efforts to re-establish it by subsequent pro-social behavior should be strongest.

### Method

#### Participants and Design

One hundred and fifty subjects (87 female) participated in the study that was conducted in the city center of Straubing, Germany. A research assistant randomly approached pedestrians by asking them to participate in the study. Subjects received a compensation of 4€ (USD 5.18) for participation and had an additional chance to win 30€ (USD 38.87).

Participants were randomly assigned to one of two conditions (product label: green/conventional) of a one-factorial between-subjects design.

#### Purchase Scenario

Participants read a brief introduction section including an imaginary shopping scenario and were asked to envision having purchased the target product, which was subsequently presented (green or conventional label). The purchase situation allowed for both increasing the external validity of the findings as well as for evoking an imbalance in participants’ moral self-concept due to either purchasing a conventional or green-labeled product.

#### Product Label

After passing the introduction section, participants were randomly allocated to one of the two product label conditions. A picture of suntan lotion that was either labeled as green or conventional was presented on a screen. In the green label condition, the product name included the word *nature* (i.e., *Lorane Nature Suntan Lotion*) that was omitted in the conventional label condition. Additionally, the green-labeled product included a picture of two leafs while the conventional product depicted a sun and a female face. We did not provide any official seals (e.g., eco labels) in any product label condition as a seal might have conflicted with the provided environmental information in the subsequent section. We conducted a pre-study (*N* = 113) confirming that the nature product was indeed perceived as more natural [*t*(111) = 5.96, *p* < 0.001].

#### Product Attribute Information

To conceal the actual purpose of the study, we included three information categories in addition to the environmental information: dermatological quality, employee security, and animal protection. Participants were told that external experts evaluated each product attribute. Evaluation was based on a point-based scale ranging from zero points (*very bad*) to three points (*very good*) and was verbally justified by a short paragraph. The product’s environmental attributes were rated as poor (one of three points) because of high CO_2_-emissions due to importing ingredients from overseas and the partial utilization of synthetic preservatives. Additionally, the packaging material was described as only moderately recyclable. The remaining attributes were evaluated with two (employee security) to three points (dermatological quality and animal protection).

In an additional pre-study (*N* = 107), we tested whether the environmental information affected perceptions of the product’s environmental attributes. The pre-study was based on a one-factorial between-subjects design (environmental information: positive/negative). The positive environmental information condition included three evaluation points and described the product’s environmental attributes as entirely positive (i.e., CO_2_-emissions due to transportation, ingredients, and recyclability). The negative environmental information condition was identical to that of Study 2. We conducted an ANOVA including environmental information as factor and participants’ ratings of the product’s environmental attributes as the dependent variable. Results revealed that participants rated the environmental attributes as significantly superior in the positive environmental information condition [*F*(1,105) = 12.66, *p* = 0.001, $\eta_{p}^{2}$ = 0.11]. We further checked whether variation in environmental information affected the perceived quality of the information. A MANOVA including the environmental information factor and participants’ ratings of how informative, objective, and credible the environmental information was showed no significant main effect of information [*F*(3,103) = 0.032, *p* = 0.992, $\eta_{p}^{2}$ < 0.01].

#### Measurements

##### Ecological motivation

Similar to Study 1, ecological motivation was measured with four items that were adapted to the domain of body care products (Cronbach’s α = 0.86) and veiled in a set of 20 motive items.

##### Self-congruity

Participants rated the extent to which they agreed that three personality traits described their actual self as well as that of a typical user of the allocated suntan lotion on 6-point scales (1 – *totally disagree* to 6 – *fully agree*). Using the absolute difference approach (Sirgy, 1982; Sirgy et al., 1991), we calculated the absolute differences between participants’ ratings regarding their actual selves and those of a typical product user. The actual self-congruity score was based on the mean absolute difference values whereby lower values indicate high self-congruity.

Personality traits used to determine self-congruity were gathered from a pre-study sample. We randomly allocated 66 participants to either express personality characteristics of a typical user of the green or the conventional suntan lotion. Based on a content analysis, we selected the most frequently expressed personality characteristics for each suntan lotion (i.e., for the conventional suntan lotion: thrifty, conventional, and family conscious; for the nature suntan lotion: environment-conscious, natural, and wealthy).

##### Product evaluation

Product evaluation was measured twice: first, after participants were exposed to the product picture (t1) and second, after receiving product information (t2). Measurement was based on a visual analog scale ranging from 0 (*very bad*) to 100 (*very good*). The respective evaluation value reflected the distance between the indicated point and zero. We aimed to examine the extent to which the provided product information resulted in a relative decrease or increase in participants’ individual product evaluations (as a function of ecological motivation and product label). Hence, we applied a relative measure assessing individual changes in participants’ product evaluations between t1 and t2. Relative changes in product evaluation were calculated by dividing the difference between the two product evaluation values (t2, t1) with the initial product evaluation value (t1). Thus, positive values indicate that participants rated the product as more positive at t2 as compared to t1 while negative values indicate that product evaluation was worse at t2^4^.

##### Pro-social behavior

After the product evaluation task, participants were informed that they had the chance to win an amount of 30€ (USD 38.87) in addition to the fixed compensation of 4€ (USD 5.18). Participants were asked to indicate how much of the 30€ they would donate to a child charity organization in case they won the additional compensation.

A descriptive pre-analysis revealed that the majority of participants tended to either donate all (30€) or nothing (0€) of the possible compensation. Confidence intervals in the respective analysis were based on bootstrapping techniques (bias-corrected, 5,000 samples) to take these deviations from normal distribution into account.

#### Procedure

At the beginning, participants completed a paper questionnaire including ecological motivation items as well as the personality items relevant for assessing self-congruity. Afterward, they were assigned to a computer and read the introduction text including the shopping scenario. Then, they were allocated to either the green or conventional label condition, reported the perceived personality traits of a typical product user and evaluated the presented product (t1). After product information was provided, participants evaluated the product again (t2) and were informed that they had completed the study. Finally, participants were asked to report the amount of money they were willing to donate to the charity organization, and were then debriefed, and compensated.

### Results and Discussion

#### Product Evaluation

First, we tested if participants’ ecological motivation and the product’s label affected participants’ product evaluations. Additionally, we controlled for effects of actual self-congruity. We conducted a moderation analysis [*F*(4,145) = 4.14, *p* = 0.003, *R*^2^ = 0.10] including ecological motivation, product label, self-congruity, and the ecological motivation × label interaction as predictor variables and the relative change in product evaluation as criterion^5^.

**FIGURE 3:**
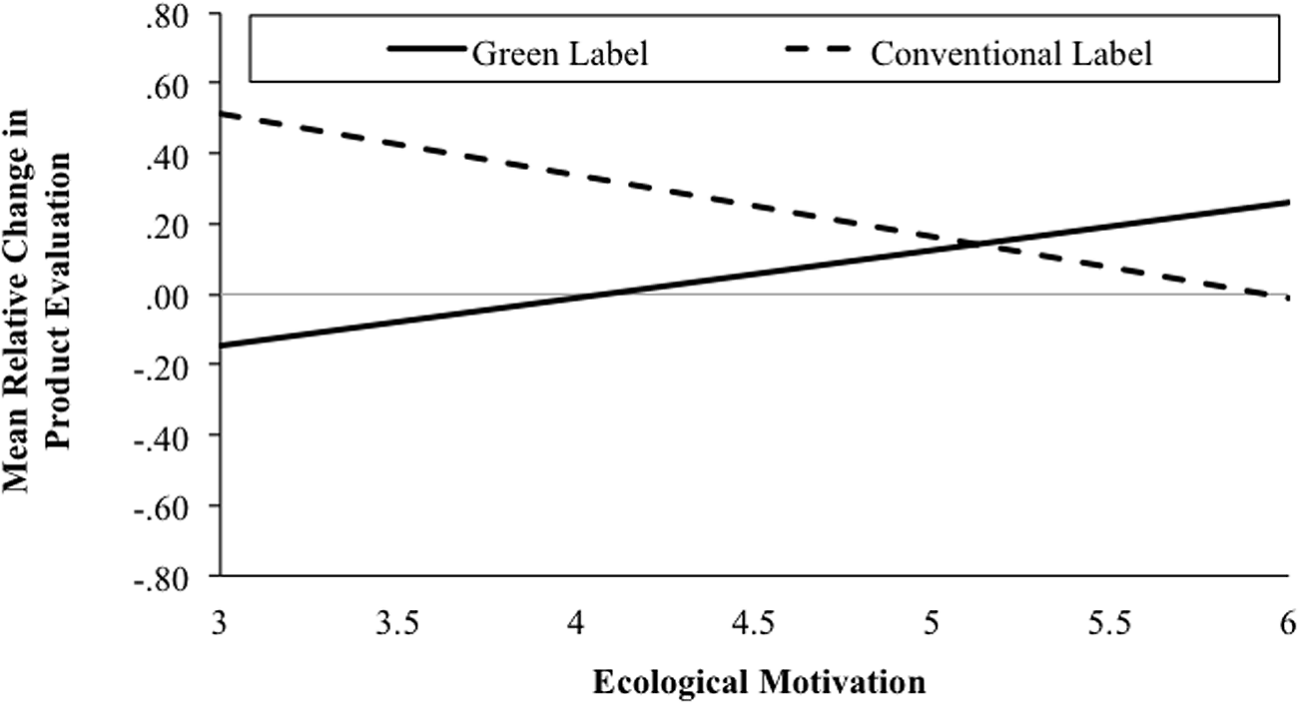
**Conditional effects of ecological motivation on relative changes in product evaluation as a function of the experimental variation (product label: green/conventional) in Study 2**.

Self-congruity influenced relative changes in product evaluations. Ecological motivation had an additional effect on the relative evaluation change score. This impact, however, interacted with the product’s label. Specifically, there was a significant direct effect of actual self-congruity on the relative evaluation change score (*b_4_* = 0.38, 95% CI [0.21, 0.54], *t* = 4.59, *p* < 0.001) showing that high self-congruity resulted in a relative decrease in product evaluations after product information was provided. Direct effects of ecological motivation (*b_1_* = –0.02, 95% CI [–0.13, 0.09], *t* = –0.36, *p* = 0.72) and product label (*b_2_* = 0.11, 95% CI [–0.12, 0.34], *t* = 0.98, *p* = 0.33) were not significant, while the interaction of both variables was (*b_3_* = –0.31, 95% CI [–0.52, –0.09], *t* = –2.83, *p* = 0.005). Confirming Hypothesis 5, the impact of provided product information varied as a function of participants’ ecological motivation and the product’s label (green/conventional). Conditional direct effects revealed a negative relationship between ecological motivation and the relative change in product evaluation in the conventional label condition (*b_1_* |*_Label__=__conv_* = –0.17, 95% CI [–0.33, –0.02], *t* = –2.18, *p* = 0.03) and a positive trend in the green label condition (*b_1_* |*_Label__=__green_* = –0.14, 95% CI [–0.01, 0.28], *t* = –1.83, *p* = 0.07). Hence, participants with high ecological motivation relatively decreased their product evaluations after negative ecological information had been provided when the product was conventional (cf. **Figure 3**). In line with the findings of Study 1, effects were reversed when the product was labeled as green.

#### Pro-Social Behavior

Second, we analyzed the effects of product label and ecological motivation on participants’ post-purchase behavior. We kept the specification of the predictor variables constant and included the amount participants were willing to donate as the criterion [*F*(4,145) = 3.80, *p* = 0.006, *R*^2^ = 0.10]. We applied the Johnson–Neyman technique to determine the value along the ecological motivation continuum, at which the effect of the product’s label transitioned from statistically non-significant to significant.

**FIGURE 4:**
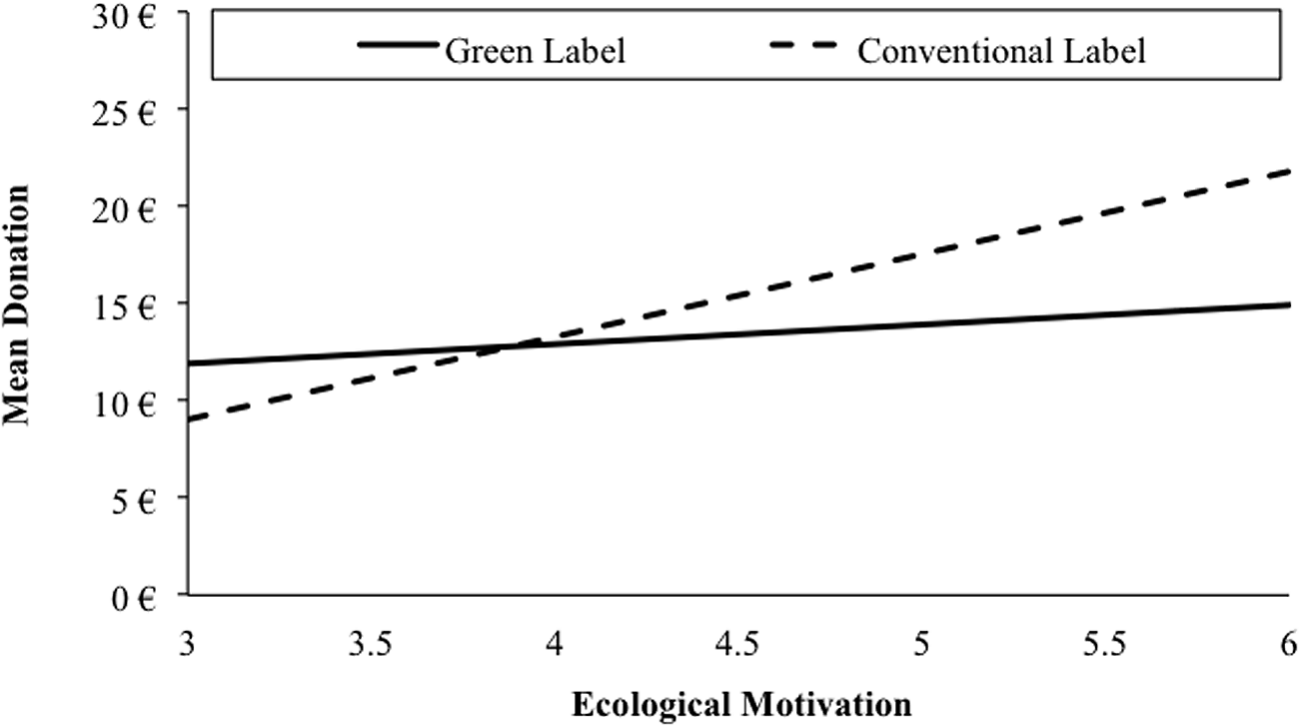
**Conditional effects of ecological motivation on the amount participants’ were willing to donate as a function of the experimental variation (product label: green/conventional) in Study 2**.

Self-congruity as well as ecological motivation had an impact on the amount participants were willing to donate. The label’s impact on donation was only evident for participants with high ecological motivation. Specifically, results showed a significant direct effect of actual self-congruity on donation (*b_4_* = –3.42, 95% CI [–6.50, –0.24], *t* = –2.24, *p* = 0.03). Strong self-congruity prior to information acquisition resulted in comparatively higher donations. The direct effects of ecological motivation on the amount donated (*b_1_* = 2.64, 95% CI [0.64, 4.62], *t* = 2.63, *p* < 0.01) reached significance. In contrast to Hypothesis 6, the direct effect of product label did not affect donation (*b_2_* = 2.89 95% CI [–1.39, 6.89], *t* = 1.34, *p* = 0.19). In line with Hypothesis 7, findings revealed a trend in terms of the interaction of ecological motivation and product label on donation (cf. **Figure 4**; *b_3_* = 3.26, 95% CI [–0.39, 7.05], *t* = 1.61, *p* = 0.08). We found that the effect of product label was exclusively significant for participants with an ecological motivation greater than 5.41 (i.e., the 65^th^ percentile of the distribution). When participants’ ecological motivation was below this value, the product label did not statistically affect their donation.

Findings of Study 2 corroborate previous research on self-congruity, showing that the perceived match between a typical consumer and one’s own personality traits strongly impacts product evaluations (Sirgy et al., 1991). In the present research, strong self-congruity was associated with higher initial product preferences, which were decreased after (negative) information was provided. It is indicated that effects of ecological motives on product evaluation were due to processes beyond previously confirmed self-congruity effects (e.g., Sirgy et al., 1991). Thus, although we controlled for actual self-congruity, ecological motivation exerted an influence on product evaluations after product information had been provided. Effects of ecological motivation varied as a function of the product’s label. As participants envisioned actually having purchased the product, the negative product information potentially threatened moral self-concepts of participants with high ecological motivation. In the green label condition, the label competed against the provided information. We assume that, under this condition, the created match established by the label served as a means to attenuate moral threats due to the negative environmental information.

Findings in terms of participants’ consequent pro-social behavior provide insights into the influence of green labels on purchase decisions. Findings showed that green label effects went beyond a mere stimulation of preference tendencies toward the labeled product when ecological motivation was high. Crucially, the cognitive match created by the green label made the purchase more morally justifiable for participants with high ecological motivation. These participants showed consistent pro-social behavior as they generally donated more than low motivated participants. When they envisioned having purchased a conventional product, their pro-social behavior was even stronger. We propose that, in the conventional label condition, the label did not provide an opportunity to cognitively attenuate threats due to negative environmental information. In accordance with moral cleansing effects, it is indicated that, in the conventional label condition, the impaired moral self-concept was restored by the subsequent donation task (e.g., Sachdeva et al., 2009).

Interestingly, the subsequent pro-social behavior was not specifically related to the environmental domain, but nevertheless effective in serving as compensation for the environment-specific moral threat. Corroborating previous research, this finding indicates that moral cleansing can occur across moral domains (Sachdeva et al., 2009; Blanken et al., 2015). In Study 3, we aimed to shed further light on the moderating function of ecological motivation with regard to purchase and post-purchase behavior.

## Study 3

In light of the findings from Study 2 showing a moderating role of ecological motivation, we aimed to replicate Mazar and Zhong’s (2010) seminal study on product labels’ (green/conventional) influence on consumers’ subsequent behavior and added the concept of ecological motivation. This design allowed for gaining deeper insights into the function of consumers’ motives as drivers of consistency as well as into adaptive processes that individuals employ when consistency is structurally inhibited.

Instead of measuring ecological motivation by means of assessing the perceived strength of domain-specific motives, we applied an alternative, behavior-based approach to capture a person’s extent of ecological motivation in Study 3. That is, we assessed the degree to which a person disregards the difficulty of a behavior in their environmental-protective engagement (see Campbell, 1963; Kaiser et al., 2015). According to the Campbell Paradigm (Kaiser et al., 2010), a person’s motivation to act in a certain way (e.g., protect the environment) becomes obvious in the face of increasing difficulties (e.g., painful sacrifices that come with an action). Why would people refrain from car use, buy seasonal produce, reuse shopping bags or endure lower temperatures at home if they were not highly motivated to protect the environment? Likewise, why would people feel bothered about selecting products with conventional labels in the course of an online experiment, thus subsequently engaging in intensified pro-social behavior, if being environmentally friendly has no relevance for the self? Thus, and in line with results from Study 2, we predicted that the products’ labels would exclusively exert an effect on subsequent pro-social behavior for participants with high ecological motivation (cf. Hypothesis 7).

### Method

#### Participants and Design

A sample of 277 subjects (162 female) – recruited via social networks, mailing lists and written calls for participation at public places – participated in the online experiment. The study was based on a 2 (product label: green/conventional) × 2 (task: purchase/evaluation) experimental between-subjects design. As compensation, one in twenty-five participants in the purchase condition were provided with the products they selected from the online store (up to 20€ [USD 24.81]). Similarly, one in twenty-five participants in the evaluation conditions won a gift certificate worth 20€.

#### Product Label

In line with the original study by Mazar and Zhong (2010), participants were assigned to one of two online stores that carried either predominantly (i.e., with a ratio of nine to three products) green or conventional products, depending on the condition. Pictures, product names, and descriptions labeled each product as either green or conventional (for the material of the original study see: Mazar and Zhong, 2010).

#### Measurements

##### Purchase decisions

As a manipulation check, we recorded participants’ choice of products in the two purchase conditions. Participants could select products of up to 20€ from the online store. The maximum number of items per product was limited to one.

##### Ecological motivation

We adopted 25 self-report items from the General Ecological Behavior (GEB) scale (Kaiser and Wilson, 2004) to estimate ecological motivation behavior-based as suggested by the Campbell Paradigm.

Typical examples of items were “I reuse my shopping bags” and “I bicycle or take public transportation to work or school.” Of the 25 items, 15 were assessed on a 5-point scale (1 – *never* to 5 – *always*) and dichotomized to match the coding of the remaining items. In line with previous similar GEB scale calibrations (e.g., Kaiser and Wilson, 2004), the Rasch Model (see Rasch, 1960/1980) served as the measurement model.

Ecological motivation levels were derived based on a maximum likelihood approach, and estimated as logits, which stand for the natural logarithm of the engagement/non-engagement ratio of a person across all items. Larger positive logit values thus reflect a more pronounced motivation. The Rasch-based person separation reliability turned out to be acceptable at rel = 0.66.

##### Pro-social behavior

Participants’ willingness to fill out additional environmental psychological questionnaires served as a measure of pro-social behavior. Specifically, we assessed the number of pages (0–11) of the additional questionnaire they filled out. This was in line with a number of other studies asking for further participation in order to unobtrusively explore open pro-social or ecological behavior (e.g., Goldstein et al., 2011; Schnall and Roper, 2012; Eskine, 2013). Furthermore, participation in environmental psychological research has been demonstrated to be a function of ecological motivation (Kaiser et al., 2011).

A descriptive pre-analysis revealed excessive skewness (1.11; *SE* = 0.15) in the distribution of the numbers of pages completed. Similar to the moderation analysis regarding pro-social behavior in Study 2, confidence intervals in Study 3 were based on bootstrapping techniques (bias-corrected, 5,000 samples) to take these deviations from normal distribution into account.

#### Procedure

Participants first filled in the GEB scale. Next, depending on experimental condition and in line with Mazar and Zhong (2010), they were either invited to select products from the online store (purchase condition) or asked to rate the store’s products one-by-one in terms of the esthetics of their design and the information content of their description, using a star rating scheme with 0 to 5 stars (evaluation condition). Then, subjects answered a list of socio-demographic questions and were probed for their willingness to fill out additional environmental psychological questionnaires, which would take them about 25 min and for which they were not offered any compensation. If they agreed, participants were directed to another online questionnaire unrelated to the present analyses. Finally, all participants were thanked and debriefed.

### Results and Discussion

#### Purchase Decisions

Group comparisons revealed no difference between conditions in the total amount of money spent in the green (*M* = 14.0; *SD* = 5.7) and conventional (*M* = 13.4; *SD* = 6.4) stores, *t*(136) = 0.6, *p* = 0.57. Importantly and in line with our manipulation, participants in the green store spent more money on green products (*M* = 11.3; *SD* = 5.8) than participants in the conventional store (*M* = 4.8; *SD* = 4.2; *t*(119) = 7.6; *p* < 0.001). Vice versa, participants in the conventional store spent more on conventional products (*M* = 8.6; *SD* = 6.2) than participants in the green store (*M* = 2.7; *SD* = 3.2; *t*(105) = 7.2; *p* < 0.001). Expectedly, participants’ ecological motivation turned out to be a positive (negative) linear predictor of the amount of money spent on green (conventional) products (*r* = 0.16, *p* = 0.03, and *r* = –0.22, *p* = 0.004, for green and conventional products, respectively).

#### Pro-Social Behavior

We next explored the effect of the predominant product label (green vs. conventional) and participants’ ecological motivation on post-purchase pro-social behavior. In line with Mazar and Zhong (2010), we additionally controlled for task effects (purchase vs. evaluation). To this end, we conducted a moderation analysis [*F*(4,145) = 5.48, *p* < 0.001, *R^2^* = 0.07] including ecological motivation, product label, task, and the two way interactions of ecological motivation × label and task × label as predictor variables and the number of pages as the criterion.

**FIGURE 5:**
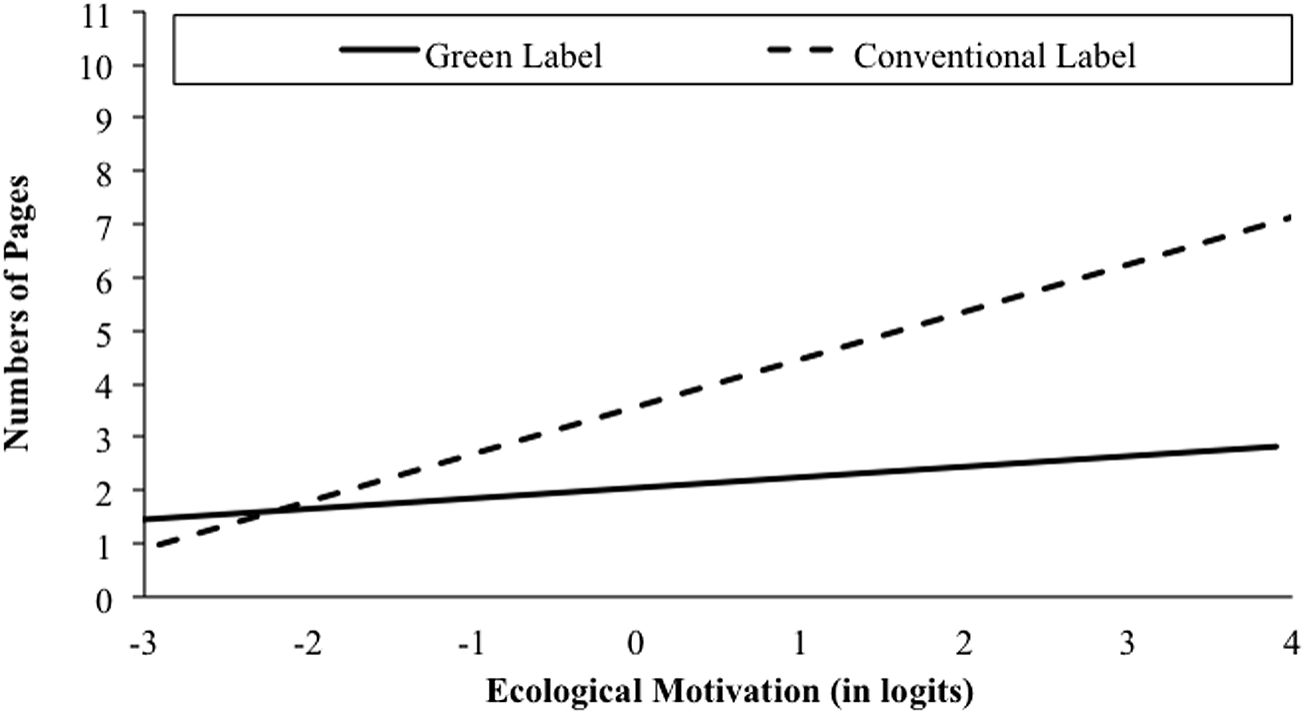
**Conditional effects of ecological motivation on the number of pages completed in the subsequent study as a function of the experimental variation (product label: green/conventional) in Study 3**.

Again we found that the label’s effect on post-purchase pro-social behavior was exclusively evident for participants with high ecological motivation (see **Figure 5**). Specifically, the regression analysis corroborated the expected direct effect of participants’ ecological motivation (*b_1_* = 0.60, 95% CI [0.09, 1.11], *t* = 2.30, *p* < 0.022). Increasing levels in ecological motivation were thus expectedly reflected in more post-purchase pro-social engagement. In addition, there was a significant direct effect of the product label (*b_2_* = 1.49, 95% CI [0.53, 2.44], *t* = 3.10, *p* = 0.002). Participants allocated to the conventional store turned out to be more pro-socially engaged, filling out a higher number of pages of the subsequent study than participants allocated to the green store. In contrast to Mazar and Zhong’s (2010) findings, the interaction of task and product label (*b_5_* = 1.31, 95% CI [–0.62, 3.24], *t* = 1.33, *p* = 0.18) did not affect the number of filled out pages. Correspondingly, the expected interaction effect of the predominant product label and participants’ motivation did not reach statistical significance (*b_4_* = 0.8, 95% CI [–0.22, 1.83], *t* = 1.54; *p* = 0.12). More importantly, however, applying the Johnson–Neyman technique, we found that the effect of the predominant product label on the number of pages filled out was exclusively significant for participants with a moderate to high level of ecological motivation (i.e., greater than –0.58, the 28th percentile of the distribution; cf. **Figure 5**). Thus, while we did not find evidence for the assumption of a moderating influence of participants’ motivation, the products’ label did turn out to be more or less irrelevant for people who were only weakly motivated to protect the environment (cf. Hypothesis 7).

In summary, Study 3 highlights the importance of product labels and individuals’ ecological motivation for predicting both, green purchase decisions and post-purchase behavioral patterns. Employing the experimental procedure of Mazar and Zhong (2010), we found that participants’ ecological motivation was a significant predictor of the amount of money spent on green products in the purchase task. Moreover, participants purchasing or evaluating conventional products subsequently engaged in more pro-social (here: pro-environmental) behavior than participants exposed to green products (i.e., moral cleansing), but only if their ecological motivation was comparatively high. Supporting our assumption that ecological motives are deeply anchored in a person’s moral self-concept, highly ecologically motivated consumers forced to act inconsistently with their motivation (i.e., in the conventional label condition) seemingly attempted to re-establish their threatened moral self-concept with intensified pro-social engagement. Thus, the match between highly ecologically motivated consumers and green-labeled products made green purchase decisions both, more likely and more morally justifiable, which is in line with the findings from Study 2.

## General Discussion

The goal of the present research was to provide detailed insights into psychological mechanisms of green labeling. Specifically, we were interested in the robustness of green label effects when product information contradicts the label’s image. To address this issue more deeply, we assessed consumers’ moral self-regulation processes in addition to purchase decisions. Thus, we examined whether green labels are effective in preventing consumers from moral threat due to the purchase. Overall, we expected green label effects on purchase decisions and post-purchase behavior to be most pronounced when consumers have high ecological motivation (see also e.g., Lee et al., 2013; Sörqvist et al., 2013, 2015b).

We hypothesized that green labels and product images would increase purchase behavior by making consumers perceive the product as compatible with their motives. This match was expected to be particularly likely when consumers have high ecological motivation. Correspondingly, our findings showed that high ecological motivation resulted in strong preferences for green-labeled products. The anticipated compatibility between green-labeled products and consumers with high ecological motivation diminished the influence of contradicting product information. Moreover, green labels protected those consumers from moral threats due to negative environmental information. The same negative product information harmed moral self-concepts of highly ecologically motivated participants, prompting corrective pro-social behavior when the product had a conventional label. Hence, our findings also contribute to research on moderators between behavioral consistency and moral self-regulation (e.g., Cornelissen et al., 2013). In Studies 2 and 3, ecological motivation substantially determined whether participants engaged in moral self-regulation processes or not.

We proposed effects to be attributable to consumers’ pursuit of acting consistently to their motives. With regard to ecological motives, this may imply adaptive product inferences and information processing when the product signals to be environmentally friendly. When the first impression based on the label is formed, available product information competes against the already established tie between the product and consumers’ motives. Supporting our assumptions, the impact of ecological motivation varied depending on whether the product signaled to be sustainable or not. When the product had a pro-environmental image (Study 1) or was explicitly labeled as green (Study 2), participants with high ecological motivation had stronger preferences for the product after product information was provided. This effect even prevailed when product information stood in sharp contrast to the product’s label.

The established link between the green-labeled product and consumers’ ecological motives was strong, even protecting highly ecologically motivated consumers from moral threats due to purchase. We assessed effects of green labels on the moral level by incorporating participants’ pro-social behavior consequent to the purchase (Studies 2 and 3). Findings support our assumption that ecological motives are deeply anchored in individuals’ moral self-concept. Participants with high ecological motivation acted, consistent with their motives, more environmentally friendly in post-decision occasions as these participants voluntarily filled out more environmental psychological questionnaire pages. Moreover, participants with high ecological motivation also comparatively donated more money for a pro-social charity organization unrelated to the environmental domain. Our findings are line with research on values (Schwartz et al., 2012), showing that the motivation to protect the environment is empirically related to other pro-social motives (e.g., caring, concern).

In our research, effects of ecological motivation on pro-social behavior were boosted when highly ecologically motivated participants were experimentally forced to choose or even merely evaluate conventional products. Apparently, consumers failing to act in line with their moral standards, dynamically adapt their subsequent behavior in order to re-establish the self. Applying a green label to products, however, protected consumers with high ecological motivation from such threats to their moral self-concept, thus diminishing the need to compensate via pro-social behavior.

Moral self-regulation was not limited to the environmental domain. Moral cleansing effects even occurred when the respective compensatory behavior was unrelated to the environmental domain (Study 2, child charity). In accordance with findings of a recent meta-analysis (Blanken et al., 2015), our results indicate that subjective moral integrity can be re-established by a set of moral behaviors. That is, moral cleansing is potentially not limited to the specific domain in which the individual has indulged in an immoral act (see also Sachdeva et al., 2009).

Our findings extend previous research on the impact of labels on purchase decisions. In line with recent findings, effects of green labels were more pronounced when consumers’ ecological motivation was high (Lee et al., 2013; Sörqvist et al., 2013, 2015b; Wiedmann et al., 2014). These preferences for green-labeled products were not diminished when we provided negative information about the labeled product. Similarly, in previous research, the willingness to pay for green-labeled products was robust, i.e., hardly affected by personal preferences such as taste when consumers had high ecological motivation (Sörqvist et al., 2013). This heightened willingness to pay for green-labeled products was not driven by social desirability (Sörqvist et al., 2013; see also: Sörqvist et al., 2015b). Correspondingly, the present research showed that label effects were not simply based on general positive preference patterns toward green-labeled products. Findings on post-purchase pro-social behavior revealed that purchasing labeled products kept moral self-concepts of participants with high ecological motivation in balance. That is, highly ecologically motivated participants tended to favor green-labeled products, but also perceived purchasing them as more morally justifiable as compared to non-labeled products. Negative information could not diminish these effects.

Findings indicate that effects of green labeling were not based on selective acquisition of product information. In Study 1, we included control questions to ensure that provided information was actually read and understood. However, direct effects of ecological motivation on purchase intentions even prevailed when the environmentally friendly image of EVs was threatened by negative environmental information. Under this condition, participants with high ecological motivation still tended to favor EVs. In line with theories on belief harmonization (Dunning, 2007) and cognitive dissonance (Festinger, 1957), these highly motivated participants potentially engaged in cognitive alignment processes to maintain the established link between the product and their motives. These cognitive processes may, for example, encompass the denegation of the diagnosticity and importance of negative product information (e.g., Ahluwalia et al., 2000). That is, although we triggered a central route of information processing (Petty and Cacioppo, 1986), the green image was nevertheless effective in stimulating positive purchase intentions when ecological motivation was high.

## Limitations

We aimed to keep the time span between measuring ecological motivation and the purchase tasks as long as possible. In addition, we presented ecological motives within a set of further motives to reduce attention to ecological items in Studies 1 and 2. We replicated findings of Study 2 by applying a validated behavior-based measure of ecological motivation in Study 3. However, we cannot rule out that the mere act of reporting ecological motivation activated motives and thus increased their impact on purchase decisions.

The information we presented in Study 1 was, to some extent, open to individual interpretations. Hence, the environmental impact of EVs was described to be primarily subject to the source of energy used for driving. In Germany, the domestic energy mix depends on the chosen energy supplier. Thus, when charging at home, consumers could individually influence the CO_2_-emissions of their EV. Such circumstances may foster the generation of counterarguments (e.g., Ahluwalia et al., 2000; Ahluwalia, 2002) and provide an opportunity for cognitively attenuating presented negative information. In Study 2, product information was linked to a specific, non-technological product (suntan lotion), thus reducing room for subjective interpretations. Ecological motivation nevertheless affected the impact of provided product information. Under these conditions, participants presumably referred to other cognitive harmonization strategies such as altering the individual relevance of presented attributes or attenuating the diagnosticity of negative information (e.g., Ahluwalia et al., 2000; Ahluwalia, 2002; Greitemeyer and Schulz-Hardt, 2003).

In Study 2, the experimental variation of product label concerned the word “nature,” which was included in the product name in the green label condition (*Lorane Nature Suntan Lotion*) and the depicted product pictures. With regard to the latter, a picture of two leafs was included in the green label condition, while a picture of a female face and a sun was depicted in the conventional label condition. We cannot exclude that these differences in product pictures also affected product evaluations. We suggest future research to minimize differences between label conditions to rule out possible confounds.

The present research provides new insights into the relationship between green labels, product information, and consumers’ ecological motivation. We assumed the current findings to be based on the labels’ ability to create a match between the product and consumers in an early stage of preference formation, prior to acquisition of information. This assumption is consistent with research showing that green labels may induce halo effects. That is, green labels may affect a variety of product attribute perceptions beyond the product’s environmental attributes (Lee et al., 2013; Sörqvist et al., 2013, 2015b). Future research is needed to gain more detailed knowledge about the processes that guide information processing in purchase decisions of labeled products. A promising approach is to systematically vary the order of product labels and product information presentation to more deeply examine the impact of labels at different stages of the decision process, as one reviewer suggested. Previous research indicates that green labels even increase the willingness to pay for the product when they are presented *after* consumers had the opportunity to taste the product (Sörqvist et al., 2013).

## Conclusion

We began this article with a striking statement that seems to reflect the current market situation: green sells. Green products increasingly flood the market and so do the number of inappropriately green-labeled products – meaning unsustainable products that are labeled as environmentally friendly. In practical terms, consumers with high ecological motivation probably constitute the primary target group for green-labeled products. In the present research, this target group was especially susceptible to green product images and labeling, which weighed heavier than contradictory product information. We hypothesized the impact of labels to be based on their potential to create a cognitive match between the product and consumers’ ecological motives. This perceived link eventually protected consumers with high ecological motivation from threats to their moral self-concept that arose when product labels did not signal environmental friendliness. When purchased products had conventional labels, consumers with high ecological motivation restored their impaired moral self by intensifying their pro-social behavior in consequent occasions.

The present findings emphasize the impact of product labels or images, which may conflict with the product’s actual properties. Classic information processing models (Petty and Cacioppo, 1986) predict that highly motivated consumers should be most likely to invest cognitive resources to reveal the product’s actual properties. As the present research illustrates, even attending to detailed information, however, does not guarantee diminishing the power of putting a label on products.

## Conflict of Interest Statement

The authors declare that the research was conducted in the absence of any commercial or financial relationships that could be construed as a potential conflict of interest.

## Footnotes

1 In the present research, we use the term “green product labels/green labels” to refer to product characteristics, which signals to consumers that the labeled product is environmentally friendly (for research using the term green products see e.g.: Griskevicius et al., 2010; Mazar and Zhong, 2010). Note that green does not refer to the color green (e.g., Schuldt, 2013). In the present research, green labels were either explicit (physical) or implicit (non-physical). The term “green label” shares large characteristics with the term “eco-label”, which has been used in previous research (e.g., Sörqvist et al., 2015a,b). However, in the present research, we refer to the more abstract term “green labels” to emphasize that the applied labels may be distinct from official seals and even merely based on implicit characteristics (e.g., product reputation).

2 Independent variables, moderators, and mediators were mean-centered prior to analyses in the present studies.

3 Due to the non-significant interaction of ecological motivation and EV purchasing price information on purchase intentions and to retain clarity, we calculated direct and indirect effects of ecological motivation on purchase intentions only at values of the moderator EV environmental information (positive/neutral/negative) rather than at values of both moderators (EV environmental and purchasing price information).

4 Relative evaluation change scores of two participants exceeded two standard deviations (*SD*) above the average. We replaced these two data points by the respective relative evaluation change score that would reflect two *SD* above the mean value (e.g., Field et al., 2012). This did not result in transitions of *p*-values of the direct and interaction effects from non-significant to significant but substantially increased the accuracy of the analysis (e.g., reduction of standard errors).

5 To ensure that potential effects on the relative change score were not influenced by differences in product evaluation at t1, we conducted an additional moderation analysis including the defined predictor variables and t1 product evaluations as criterion. Importantly, neither ecological motivation (*b_1_* = –1.49, 95% CI [–4.67, 1.67], *t* = –0.93, *p* = 0.35) nor the interaction of ecological motivation and product label affected participants’ ratings at t1 (*b_3_* = 4.05, 95% CI [–2.33, 10.46], *t* = 1.25, *p* = 0.21). Direct effects of product label (*b_2_* = –9.46, 95% CI [–16.27, –2.65], *t* = –2.75, *p* < 0.01) and actual self-congruity (*b_4_* = –8.91, 95% CI [–13.73, –4.08], *t* = –3.65, *p* < 0.001) were significant. High self-congruity and the green product label resulted in comparatively more positive evaluations.
