# Supplementary material for: The power of putting a label on it: green labels weigh heavier than contradicting product information for consumers’ purchase decisions and post-purchase behavior
Source: Front Psychol. 2015 Sep 23;6:1392. doi: 10.3389/fpsyg.2015.01392 (PMC4585300; doi:10.3389/fpsyg.2015.01392)
Supplement: Supplementary file 1 [file Data_Sheet_1.DOCX]

**Appendices**

Appendix A

**Table A:** Wording of items measuring ecological motivation, perceived matching, and purchase intention in Studies 1 and 2.

| Scale | | Item |
| --- | --- | --- |
| Study 1 | | |
| Ecological motivation | | |
|  | It is important to me that I drive a car that emits little CO_2_. | |
|  | It is important to me that I harm my direct environment as little as possible by driving a car. | |
|  | It is important to me that my mobility is environmentally friendly. | |
|  | It is important to me that I cause as little CO_2_-emissions as possible by driving a car. | |
| Perceived matching | | |
|  | The necessary charging time of an EV matches my mobility needs. | |
|  | The range of an EV is sufficient for my mobility needs. | |
|  | The environmental attributes of an EV match more with me than those of a conventional vehicle. | |
|  | The CO_2_-economy of an EV only matches with me, if it is charged with 100% renewable energy. | |
|  | The purchase price of an EV corresponds to the price I would be willing to pay for a car. | |
|  | The performance of an EV (horse power, acceleration, maximum speed, etc.) meets my demands on a car. | |
|  | The charging costs of an EV match my price expectations. | |
| Purchase intention | | |
|  | I can imagine that my next car will be an EV. | |
|  | I can imagine buying an EV within the next 5 years. | |
|  | If I buy a car in 5 years, I could imagine it being an EV. | |
|  | Study 2 | |
| Ecological motivation | | |
|  | It is important to me that the production of my body care products causes as little CO_2_ as possible. | |
|  | It is important to me that the transport and the production of my body care products strains the environment as little as possible. | |
|  | It is important to me that the impact of my body care products on the environment is as small as possible. | |
|  | It is important to me that my body care products are based on sustainable materials. | |
| *Note.* In the study, items were presented in the participants' native language (German). | | |

Appendix B

**Table B:** Summary of EV attribute information presented in Study 1. EV environmental and purchasing price attributes were experimentally varied between participants, based on a 3 (EV environmental information: positive/neutral/negative)× 2 (EV purchasing price information: moderate/high) experimental between-subjects design. Information about the remaining EV attributes was kept constant across conditions.

|  | EV attributes | Presented values |
| --- | --- | --- |
| Purchasing price | |  |
|  | moderate/high | 25,000€/35,000€ |
|  |  | (USD 32,860/46,000) |
| Charging costs (for 100km) | | 5 € |
|  |  | (USD 6.57) |
| Maximum speed | | 165 km/h |
| Horse power | | 130 PS |
| Range | | 180 km |
| Charging time | | 7 h |
| CO_2_-emissions (g CO_2_/km)^a^ | |  |
|  | positive/negative/neutral | 0/175/225 |
| Note. ^a^EVs' direct CO_2_-emissions are zero but the indirect (total) CO_2_-emissions depend on the generation of the energy used for driving. | | |
|  |  |  |
|  |  |  |
